# Facebook as an arena for peer support?—Knowledge exchange and normative illness narratives about stroke

**DOI:** 10.1177/20552076251376274

**Published:** 2025-09-09

**Authors:** Lill Hultman, Ulla-Karin Schön, Fredrik Sandman, Mikael Åkerlund, Jeanette Nelson, Malin Tistad

**Affiliations:** 1 Division of Family Medicine and Primary Care, Department of Neurobiology, Care Science and Society (NVS), 7671 Karolinska Institutet, Huddinge, Sweden; 2 Division of Social Work, School of Social Sciences, Södertörn University, Huddinge, Sweden; 3 Division of Social Work, 7675 Stockholm University, Stockholm, Sweden; 4 School of Health and Welfare, Dalarna University, Falun, Sweden

**Keywords:** Peer-to-peer online support, stroke, recovery process, normative discourses

## Abstract

**Background:**

Experiencing a stroke disrupts the expected life course and can negatively impact cognitive, emotional, and physical functioning, making it a major source of disability globally. To support recovery, individuals can seek help by sharing their experiences in peer-to-peer support communities.

**Objective:**

The objective of this study was to use discourse analysis to examine how members of a peer-to-peer online support community for stroke survivors construct their understanding of the condition and use narratives to reconstruct their identity into different subject positions.

**Methods:**

The study context was a private Swedish peer-to-peer online support community created and administered by middle-aged people with lived experience of stroke. Data was collected through structured protocols that captured the content of posts and comments (*n* = 397). The analysis has followed the principles of Laclau and Mouffes discursive framework.

**Results:**

Our findings suggest that identity struggles revolve around looking ahead and negotiating normative expectations with oneself and others. We identified five subject positions: survivor, pathfinder, mentor, struggler, and outsider. These positions highlight the plurality and fluidity of post-stroke identity processes. Members often shifted between these roles based on changes in health, emotional state, or community interactions.

**Conclusions:**

The diversity of subject positions demonstrates that stroke recovery is not a uniform process, emphasizing the importance of recognizing identity work as a core aspect of post-stroke adaptation. The findings highlight the need for more flexible, patient-centered services that accommodate diverse narratives, including those shaped by long-term disability, emotional trauma, and shifting life priorities.

## Introduction

This study examines how members of an online community for individuals with personal experience of stroke construct their understandings of the condition and use narratives to reconstruct their identities on a peer-to-peer online platform. Experiencing a stroke often disrupts the conventional progression of the life course, challenging the expected timing of normative life stages.^1^ A normative life course typically implies a sequence of milestones, such as graduating from school, leaving home, securing employment, finding a partner, and having children. These structured trajectories are referred to as cultural scripts.^2^

In stroke recovery, particularly for many young stroke survivors, life cannot be put on hold. Compared to retirement-aged individuals’ younger people are in a different stage in their life course trajectory, where many are still gainfully employed parents.^3,4^ People with lived experience of stroke described a loss of role, loss of belonging, loss of control, and the loss of ability as part of everyday life.^5,6^ Many also described dependencies on others and a fear of being perceived as a burden of care,^7^ increased reliance on family members,^8^ and financial hardship.^9,10^

A discourse analysis of the impact of early stroke on identity shows that participants who were under the age of 55 years, were sensitive towards negative responses from carers, which they tried to mitigate by downplaying the negative consequences of stroke.^11^ They were also attentive to whether their accounts were perceived as persuasive or believable by carers, which displays sensitivity to interactional aspects of stroke, which provides an important demonstration of the context-dependent nature of identity constructions and how carers made it difficult for them to maintain a positive sense of self in the presence of their carers.^11^ To explore whose understandings and rules of relevance determines the rehabilitation processes Bendz explored how stroke survivors under 65 years deal with rehabilitation and understandings of stroke.^12^ In this study the biomedical discourse of stroke survivors overlap with healthcare staff since there was a shared focus on the physical rehabilitation process. However, there were also differences. For stroke survivors it was important to understand stroke recovery as a state, that is, to portray themselves as individuals who filled important roles in society which they wanted to reclaim. Health care staff had a more limited perspective of recovery, with focus on regaining physical and cognitive capacities, which made them focus on the impairment (the fragmented self) instead of a more holistic understanding of stroke survivors’ identity.

Peer support has traditionally been conducted offline in face-to-face groups. Offline and online support groups have different strengths and weaknesses.^13^ The role of in-person peer support groups for adult stroke survivors has been evaluated. A systematic review found they offered a space for shared experience, social comparison, vicarious learning, and mutual gain.^14^ Peer support groups for adult stroke were a place where people shared knowledge, experience a sense of belonging and found a purpose in mentoring each other.^15,16^

Digital support groups have the potential to promote healthy lifestyles and better mental health.^17^ Social media provides an opportunity for users to engage in different communities which offer a variety of experiences, such as providing and receiving support.^18^ Virtual support groups provide great access and convenience since they can reach people who otherwise would be limited in ability to receive support. Predictors of participation in online peer support are: (a) limited access to support within traditional social networks, (b) living with health-related stigma, (c) perceived similarity and credibility of support providers, (d) convenience and other features of computer-mediated support.^19^ People are more willing and feel more comfortable in sharing sensitive information or asking sensitive questions in an online community.^20,21^ Online peer-support shares positive aspects that are found in offline peer support groups; social connectedness,^22^ helps to cope with stigma and challenges in everyday life,^23^ facilitate insights into health care decisions, empowerment, and recovery processes.^23–25^ Connecting with people who have gone through a similar experience can be a helpful way to adjust to a new life situation.^26^ Online communities provide environments in which people with similar health concerns can interact and exchange information about self-care of long-term conditions^27^ such as mental health, alcohol addiction and self-harm.^28–30^ The shared space allows members to influence usage of health care resources and facilitate illness self-management.^31^ It also enables members to express psychological (e.g., belonging), social (e.g., norms of reciprocity) and functional (e.g., access and convenience) needs.

For some peer-to-peer, online communities for people with stroke may be illustrative of how different discourses can enable people to construct social identities, and how knowledge which both reflects and contributes to shaping our social world. In this way discourse analysis underlines how, different members engage with different types of discourses and how those different discourses enable people to construct and negotiate their identity in a closed online community that was established by and for people with first-hand experience of stroke and to a lesser extent their family members. We are familiar with the challenges that people face in relation to self, that is, loss of role,^32^ and others, being perceived as a burden of care.^7,8^ In addition, many experience financial hardship.^9,10^ However, we need more knowledge about how recovery processes are experienced and what characterizes people's identity construction in these recovery processes.

In this article, we use discourse analysis to explore how members of an online community for people with lived experience of stroke, create their understandings of stroke and utilize narratives to reconstruct their identity.

## Discourse analysis

For White and Epston^32^ persons give meaning to their lives and relationships by storying their experience.^32^ ^p. 13^ A discursive approach treats illness attributions as being constructed by speakers in the situated context of accounting for themselves to other people.^33^ Stainton-Rogers points out that people's health explanations are much more complex and flexible since they are constructed out of “culturally available discourses” and vary according to situational demands.^34^ The term “self-narrative” refers to an individual's account of the relationship among self-relevant events across time in which we establish coherent connections among life events.^35^ In a digital support group such as a Facebook (FB) community for stroke survivors, self-narratives can be viewed as forms of social accounting or public discourse, in which construction of self-narratives are open to continuous alteration due to interaction between members to either sustain, enhance or impede various actions. In this sense self-narratives emerge within processes of ongoing interchange between different actors. In a broad sense they serve to unite the past with the present and to signify future trajectories. In FB communities, there is a continuing negotiation of narrative identity where the legitimacy concerning self-identity must be confirmed by other members, that is, narrative validity depends on the affirmation of others.^36^ This reliance of others places the narrator in a position of interdependence, a network of reciprocating identities. Accounts of different stories are examples of different discursive practices in which people produce social and psychological realities. In this context different discourses exemplify different perspectives and interpretations of reality. Discourses change over time; thus, the discourse we assume changes the way we perceive our reality and its inherent meaning. The overall idea of discourse theory as presented by Laclau and Mouffe is that everything changes over time and no social phenomena is ever “finished.”^37^

Discourse analysis has a social constructionist foundation in which theory and method are treated as a unit.^38^ Discourses are specific ways of talking about and interpreting the world, and in doing so, they help shape our understanding of it. Discourses are both constructed by society and, at the same time, construct the reality we experience.^39^ Discourses highlight certain actions or behaviors that are considered possible and relevant in particular situations, which means that the way we understand these situations can have significant social consequences. Through discourse, individuals are offered subject positions, meaning ways of positioning themselves within the conversation. A subject position is a discursive role that, like the discourse itself, is open and flexible.^38^ For example, in the context of stroke recovery, the discourse surrounding rehabilitation might position the stroke survivor as either a passive patient in need of constant care or as an active participant in their own recovery. In some discourses, the survivor may be expected to focus on medical treatments and rely on healthcare professionals, while in others, the focus may shift toward self-empowerment, with the individual taking an active role in setting rehabilitation goals and participating in activities designed to promote recovery. The subject position of “stroke survivor” can thus shift depending on the discursive framework, influencing both how recovery is approached, and the expectations placed on the individual.

Discourses are socially constructed and reshaped through interactions with other discourses, which means that there is a struggle over meaning within these discursive exchanges.^37^ This process emphasizes what enables discursive practices to take place in specific contexts.^39^ Laclau and Mouffe^37^ refer to key moments of meaning-making within discourse as nodal points. These nodal points serve as central anchors around which other elements of the discourse are organized. The meaning of each element, or signifier, is shaped by its relationship to these nodal points. Within any discourse, nodal points act as “privileged signifiers that fix the meaning of a signifying chain, which are also referred to as chains of equivalence.^37^ ^p.112^

Discourse is not only about what is explicitly stated but also about what remains unsaid. It exerts a normative effect, determining what is considered acceptable to say or write in a given context.^40^ This is particularly evident through exclusionary processes, where certain ideas or practices are silenced or prohibited. For instance, in the context of stroke rehabilitation, the discourse of what is relevant regarding recovery has been reported to prioritize the perspectives/discourses of health care professionals^13^ which might position them as central and non-negotiable.

Meanwhile, alternative approaches—such as patient-led recovery strategies or holistic care—might be marginalized or excluded from mainstream discussions. The exclusion of these alternative practices doesn’t always require an explicit prohibition but is often maintained by the dominant discourse's framing of what constitutes “valid” recovery practices. This regulation of what is considered legitimate or possible within stroke rehabilitation serves as a form of discursive control, shaping both professional practices and patient experiences.^40^

The discourse analysis in this study is inspired by the theoretical framework developed by Laclau and Mouffe.^37^ The focus is primarily on identifying nodal points, subject positions, and broader discursive frameworks. The overarching aim of this study is to explore how members of an online community for people with own experience of stroke, create their understandings of stroke and utilize narratives to reconstruct their identities. More specifically the following questions will be studied: Which subject positions are represented in a private FB community for people with stroke, and how can these positions be understood in terms of reconstructing identity after stroke? Which norms and societal structures can be related to such positions?

### Constructing recovery narratives

Because this paper is concerned with stroke survivors’ construction of identity in their recovery process rather than the nature of stroke *per se*, it is important to identify discourses that relate to experiences of stroke. The ways in which language is used in specific contexts so-called discursive practices construct certain versions of the world and rejects others.

Interpretive concerns with illness have been of long-standing interest in the sociology of health and illness. Classic works like Bury^41^ have contributed to the analytic vocabulary of biographical disruption. Kaufman and Becker^42^ pioneered qualitative investigation into stroke, with an emphasis on the construction of stroke recovery. This pattern is remarkably similar throughout the stroke literature.^43^ Following any life-changing event, people's sense of self is fluid. The social and psychological effects of the sudden transition from being able-bodied to disabled effects this fluidity having to cope with a wide range of physical, psychological, social, and sexual impairments. Personal identity is based on two core dimensions: one that grounds personal identity in autobiography along with basic needs, wishes, and intentions, and the other that sees moral traits, more specifically one's sense of interpersonal responsibility, as the significant part of personal identity as recognized by others. In the narrative approach each *Self* is considered the product of a narrative, co-created in relational contexts and in dialogue with other internal *Selves.*^44,45^

To explain the experiences of living with long-term conditions such as stroke, chronic illness models are predominantly used. In these models the redefinition of life and identity becomes a major task.^46,47^ Chronic illness trajectory framework consists of several phases reflecting the endless and continuous process of revising the experience, coping, and adapting to the new and stressful situation. Illness narratives can either focus on restitution, quest, or chaos.^48^ In the restitution narratives, the emphasis is on recovery and that one must concentrate on getting well by following professionals’ advice concerning treatment options, and possible outcomes. The quest story expresses acceptance of the illness and its consequences. The focus is on gaining a new purpose in life, being a “re-born.” The quest stories often contain three stages of a journey described by Campbell^49^: departure (a) (early body signs of something not being right), (b) initiation (symptoms become too obvious to be mistaken, followed by “a road of trials” to obtain the correct diagnosis, atonement, or gaining important self-knowledge), and (c) return (being “marked by the illness”). The chaos narrative expresses the belief that life is never going to get better. These narratives can be threatening, anxiety-provoking, and concerned with emotional battering. Focus is failure or being incapable of taking control of one's life or illness. This could also include experiences of lack of support, comfort, or recognition from other people.^49^

### Rehabilitation processes and expectations of work return

In “modern Western societies,” people are increasingly expected to shape their lives by making individual choices, in which the “ideal citizen” is supposed to be independent and productive.^50,51^ The Swedish discourse on stroke rehabilitation is governed by laws and guidelines originally formulated in connection with the 1992 rehabilitation reform that introduced the concept of work-life-oriented rehabilitation. A fundamental context for professional stroke rehabilitation practice has been provided by the publishing of extensive political guidelines and research on how professionals can provide evidence-based and client-centered rehabilitation of high quality. Early and coordinated rehabilitation expresses an overriding societal discourse that emphasizes self-sufficiency through paid work.^52^ A discourse that presupposes a certain embodiment: the functional and able body, which makes the impairment and the disabled body a subordinate position.^53,54^ Early and coordinated rehabilitation is imbued with arguments for early interventions and fast processes through rehabilitation services.^52^ This discourse aligns with neoliberal ideology, in which the fundamental idea is to minimize public costs.^55^ In stroke rehabilitation patients are expected to participate actively and to take responsibility for his/her situation and problem. This implies a request for individualization of the services, to accommodate the neoliberal norm.^56^

### Study context and procedure

The studied online stroke community was established on FB in 2018 and is administered by four people with lived experience of stroke. The purpose of this Swedish online community is to create a safe space where members can support each other, by sharing experiences, engagement and discussing relevant topics, and establishing new friendships. When we did our data collection in the online community between November 2021 until January 2022, it consisted of 2900 members. Most of the people participating in this community were middle-aged people with lived experience of stroke, and to a lesser extent their family members (partners, siblings, and adult children).

To gain access to what members shared in this private FB community for stroke survivors, contact was established with the group's main administrator who was also the founder of the community. They acted as representatives for all group administrators (*n* = 4) and ensured that the others had the opportunity to ask questions about the purpose and procedures related to the study. When there was an agreement among the group administrators regarding access to the online community, we wanted to make sure that there also was an opportunity for members to receive information and ask questions about the study. For the administrators, it was important that members in the private community felt secure that they would not be identified by people outside of the community, since that could prevent them from sharing their experiences, thoughts, and emotions. Initially, the main administrator posted information about the study that was visible and easily accessible on the community's website. The information consisted of one brief and one more extensive letter of information about the study, as well as contact information for the responsible researchers (MT and LH). In addition, we made a film so that the members of the community had the opportunity to receive audible information as a complement or substitute to the written information.

Interactions between members were captured in structured protocol, that is templates that were specifically developed for this study by two academic researchers and three community researchers with lived experience of stroke (FS, MÅ and JN). The templets describe the content of FB posts and comments made by different members, both people with lived experience and family members. The templets specified the following items: (a) the author of the post (person with lived experience of stroke/family member/unknown), (b) the author's sex (man/woman/unknown), (c) the main focus of the post (the person with lived experience of stroke/the family member/unknown), (d) type of post (story/question/photograph/film/invitation to an event/inspirational quotes.

The first set of data was gathered for three consecutive months (December 2021 to February 2022) (see table 1). During these months all FB posts including comments that were posted in relation to the original post were included in a template (see Appendix in the online supplementary materials).

**Table 1.** Character traits of Facebook posts.

Month	Total amount of FB posts	Posts made by women	Posts made by men	Posts made by female family members	Posts made by male family members
December	98	66	32		
January	158	119	39	28	5
February	141	110	31		

There was a total of 397 posts, of which 33 were written by family members. Most of the posts were written by people with lived experience of stroke of whom the majority were women. In the first month 98 posts were noted, in the second month, 158 posts and in the third month 141 posts. In order to nuance identified subject positions and discourses, we decided to read FB posts and comments for an additional 3-month period (March to May). Only a few more posts (*n* = 9) were added between March and May 2022, as most posts during this period did not contain information that modified the identified subject positions.

The protocols (templates) were mainly filled in by the first author (LH), and then parts of the protocols were discussed in pairs, where an academic researcher collaborated with one of the community researchers.

The posts were written in Swedish and, in addition to text, they consisted of images such as photos and film clips. Sometimes the posts combined text with images or film clips. Some members only communicated through images, while others preferred the written word.

Posts were commented on with written responses; likes, emojis, GIFs, or a combination of text and pictures/symbols. A thread often consisted of a combination of stories and questions. If the original post was a question, the comments either consisted of answers, follow-up questions, or additional narratives where the commentator recognized him/herself or wanted to express validation or appreciation for what had been expressed.

### Researcher characteristics and reflexivity

Together, the authors represent a breadth of interdisciplinary and experience-based knowledge. The research group comprises medical social workers (LH and U-KS), a physiotherapist (MT), and community researchers (MÅ, FS, JN). We reflected on our roles in relation to the study population, considering our experiences in research and working with individuals with acquired brain injuries. We also examined how our values and perspectives are influenced by societal discourses. Our work in healthcare and the community has heightened our awareness of the discourses surrounding health and illness. Reflecting on our experiences with community researchers has helped ground our analysis.

### Ethical considerations

Informed consent on behalf of the members in the FB community studied was given by the group administrators. Before we initiated the study, we approached the main administrator and provided information about the purpose of the study, procedures, dissemination, as well as potential risks and benefits of participating in the study. This resulted in the main administrator discussing the implications of participating in a study with the other administrators, and came back to us, asking for clarification regarding information about data collection, usage and storage of data before giving their informed consent. To enable members in the FB community to ask questions or attend information meetings, we asked the administrator to post information about the study. In addition, we asked the administrator to pin information about the research study which contained two different letters of information, a short version and a long version. Before commencing the study, we held two digital information meetings in November 2021. To maximize participation, we scheduled the meetings twice on the same day and invited all group members. An academic researcher, a community researcher, and the main group administrator attended the information meetings. During these sessions, we explained the purpose and design of the study and provided details about the overall research project. At the end of the meetings, attendees had the opportunity to ask questions and share any thoughts or reflections that arose from the information presented. The attendees responded positively to the study. In March 2022, we held two additional information meetings. To enhance transparency, we also created a film that conveyed the same information presented during the meetings.

We protected the privacy of the members by using structured protocols that described topics that were shared in the group. In the protocols we omitted terms and phrases that could identify the members. We obtained ethical approval for this study in 2018 and in 2021 (Dnr 2018/407-31 and 2021-03548) from Etikprövningsmyndigheten [the Swedish Ethical Review Authority]. The ethical dilemma of not putting personal integrity at risk while still presenting relevant and necessary information concerning FB posts was raised and discussed within the research group. Since we had entered a private group and we could not safeguard the members’ anonymity if we quoted the original posts, hence we extracted information about posts and comments by utilizing the structured protocol where we described the content of the original post and comments. To protect personal integrity and avoid revealing any individual's personal information, the following measures have been implemented: (a) gender identities connected to individual posts referenced in the manuscript have been removed; we use “s(he)” or “they” instead, (b) locations have been omitted. There is always a risk of unintentionally distorting the contents of posts and comments, but since we still have access to the group, we had the chance to go back to the post and comments to make sure we had not added or subtracted anything.

The intimacy that can arise in this type of environment, which is usually considered because of relative anonymity, has sometimes been described as reinforcing the notion that it is a private space.^57^ Through FB's structure of the threads, it was possible to both get an immediate response to posts or later since the posts remained in the feed.

### Analysis

The first step in the analysis was to consider posts and comments as statements, and as such, as objects of analysis, and read through all the extracted protocols to identify statements to which specific discourses are connected. To identify different subject positions that were experienced by members who had their own experience of stroke, extracted posts were read and reread. To operationalize the analysis, we relied on Laclau and Mouffe's framework and key concepts; nodal points, subject positions and discursive frameworks.^37^ The key concepts were connected to and defined by so-called chains of equivalence in which each chain of equivalence belongs to a central point or master signifier.^39^ For example, expressions such as, “one who has cheated death” and “they try to beat what they can’t defeat,” became part of an equivalence chain that resulted in the subject position, “stroke survivor,” which in our study was the so-called, master signifier.^37^ First, all expressions related to different types of struggles were identified. These expressions refer to actions, feelings, and conditions that could be signs of struggling. Those expressions that were related to each other were then put together and we discovered that they created five chains of equivalence that created five central subject positions. In addition, the equivalence chains and corresponding subject positions constituted two different arenas: the struggle with rehabilitation and the struggle with identity.

## Results

In the following, we will elaborate each subject position, how these positions expressed the conditions and emotional needs associated with the subject positions, and how these positions reflected the context they operated within. From statements and narratives described in the protocols (see Table 2), five central subject positions with corresponding equivalence chains were identified. The five subject positions were gathered under two major positions: (a) struggles with recovery and work rehabilitation and (b) struggles with self-care and reconstructing an identity. The classification of these identified positions cannot be regarded as mutually exclusive.

**Table 2.** Equivalence chains and subject positions.

Equivalence chains	Subject positions	
Stroke incidents, grateful for being alive, getting a second chance to live one's life, remembering stroke anniversary/ “survival day”, being a fighter, celebrating life, focusing on the positive, making progress with physical rehabilitation and becoming more independent.	Survivor	Focus on the positive aspects of life, embodies the fighter, dedicated to making progress with physical rehabilitation and becoming independent.
Advising about medicine and other care-related issues, sharing examples of everyday life rehabilitation, suggesting strategies, answering questions, providing information about rules and regulations concerning work-related rehabilitation, and sharing experiences of work-related rehabilitation.	Mentor	Focus on sharing personal experiences and supporting others. Providing information and advising about coping strategies.
Sharing information about everyday life activities, asking for physical exercises, asking about symptoms, acquiring new skills, asking questions, reflecting on previous life choices, and having a new perspective on life, making different choices, and comparing oneself with others.	Pathfinder	Focus on personal growth; the willingness to learn and acquire new skills. Reflecting on past choices and embracing new perspectives.
Having difficulties in coping, feeling lost, experiencing helplessness, depression, brain fatigue, experiencing despair and hopelessness, anger, and resentment, being scared.	Struggler	Focus on struggling with adversity. It involves difficulties in coping. Experiencing despair and hopelessness instead of resilience.
Feeling restricted and isolated, sense of failure, feeling frustrated, feeling worthless, misunderstood, not being listened to, experiencing devaluation, loss of friends and social networks. Withdrawing from the world and focusing on negative aspects such as the loss of bodily functions and diminished social networks.	Outsider	Focus on experiences of feeling and being misunderstood, devalued and socially isolated.

### Survivor

A survivor is someone who overcomes the challenges of a stroke. They are mentally strong, committed to physical rehabilitation and achieving independence. The survivor narrative often unfolds chronologically and provides a picture of what people's lives looked like before the stroke occurred, followed by a description of the event itself, its aftermath including hospitalization, and the road to recovery at home and in the local community. In these narratives having a stroke is characterized as an overwhelming and sometimes incomprehensible life event, a situation that most people cannot perceive or imagine before it has happened to them. Being a survivor implies overcoming hardship and finding a way to successfully adapt from a stroke. It includes struggling with physical impairments, everyday life rehabilitation, returning to work, or gaining access to illness benefits or disability pension. The survivor narrative contains elements of the “success story.” For example, a protocol that describes the experience of a person with lived experience of stroke:

They talk about their rehabilitation process which they struggled through without support from employers who did not believe in their ability to recover. At the same time as it made them angry, sad and disappointed, it awakened their fighting spirit. Although they have been close to giving up, they have managed to hold on and get their old job back. They are glad that they did not get an early retirement. (Protocol 15, December)

A similar “success story” is written by a family member:

They had a massive stroke a year ago, which initially resulted in them needing a wheelchair. However, they have since regained the ability to walk. (Protocol 99, January)

Sometimes the struggle amounts to succeeding in achieving short-term goals such as tolerating setbacks and not giving in to negative thoughts, as well as long-term goals such as achieving a meaningful life after having a stroke. A part of being a survivor is inspiring others to keep up “the fighting spirit.” Several members write encouraging comments about persevering:

A member shares a video of themselves walking, revealing a slight sway in their movement. Commenters call them “fighters” and praise them for their progress. Some people express a desire to move like them. (Protocol 73, January)

Many people express gratitude for having survived. In remembrance of the day that the initial stroke occurred some people pay attention to what refer to as their “stroke anniversary” or “survival day”, which is exemplified in the following protocol:

A member expresses gratitude for the support received from both pets and people during their recovery process. (Protocol, 126, January)

In sum, the position of “survivor” reflected both society's expectations of recovery, as well as the expectations people with lived experience of stroke have on themselves.

### Pathfinder

Being a pathfinder involves navigating a transition phase, allowing individuals to explore new ways of being and deepen their self-awareness. It's about adapting to new situations, normalizing habits, and finding routines in everyday life. Understanding and accepting new circumstances can take time.

Members share, reflect on, and compare their current life situations with their lives before the stroke:

It has been three years since s(he) had a stroke and much has changed. Reflecting on this journey, s(he) has realized how different life has become. One of the biggest changes is her/his job situation. S(he) left a demanding job and returned to something s(he) used to enjoy. (Protocol 83, December)

They compare their current situation, with how it was 6 months post-stroke. Back then, they had to rest the same amount of time as an activity lasted. Today, they only need to rest once a day. However, navigating the subway with all its sensory impressions is still challenging, and they avoid social gatherings with large crowds. (Protocol 135, January)

As a pathfinder, it is crucial to share experiences with others and seek advice from those who have faced similar situations or are currently navigating them. This exchange of knowledge and support fosters a stronger, more informed community:

S(he) shares that she/he had a stroke one month ago, leading to brain fatigue and sleep difficulties. Despite several sleep medications s(he) has not found relief. s(he) wonders if anyone else has faced similar challenges. (Protocol, 86, December)

Additionally, feeling validated by other members, recognizing oneself in others’ stories, and sharing everyday life experiences become crucial parts of the normalization process, as exemplified by comments on posts:

When someone shares a photograph of a home cooked meal. Other members comment on the photograph, saying it looks delicious. Someone mentions that it brings back memories from the past. (Protocol 16, February)

### Mentor

A mentor is a constructed position of a person who has had a stroke some years ago, has been a member of the community for some time and whose advice is considered legitimate and credible. Members who have been part of the community for a long period can assume the role of mentor by informing new members of group rules but also directing attention to the value of sharing experience-based knowledge by posting questions and sharing experience-based knowledge. The role as mentor is primarily filled by individuals with lived experience of stroke.

Being a mentor is about supporting, encouraging, and validating the progress of others and it also revolves around providing hope when others are stuck in feelings of hopelessness. It is about inspiring others, allowing them to dare to dream and make plans. Mentors encourage members (both people with lived experience and those close to them) to ask questions and share experience-based knowledge which is exemplified by the following post:

The spouse shares his/her struggle to get the spouse to participate in various activities, as he/she refuses everything. He/she wonders how others handle this situation. He/she receives numerous responses from other members. They comment that it can be challenging for people who had a stroke to respond. People also acknowledge that it can be a tough adjustment for loved ones too. (Protocol 134, February)

Advice could also be provided as a direct response to a question, which often revolves around practical issues:

They ask about experiences with driving. They feel angry and sad about struggling with tasks that were easy to do before having a stroke. Another member responds that the ability to drive has returned. (Protocol 50, January)

By sharing their experience-based knowledge, mentors help other members who still are struggling to find their way (pathfinders), either people who recently had a stroke and their family or people who are a bit further down the path trying to adapt to and accept their new life situation.

In addition, a mentor can suggest coping strategies and share information about what has helped them to increase their well-being. Advice often tends to mirror societal norms about how to manage everyday life, that is, to take responsibility for your own health and prioritize taking care of one's overall health, both physical and mental aspects. In a post a mentor shares a picture with an inspirational quote that specifies a set of “life rules”:

1. Make peace with your past.

2. Don’t care about what others think of you

3. Allow things to take time.

4. The healing process takes time.

5. You are responsible for how you feel.

6. Stop comparing your life to others.

7. Stop overthinking. Smile.

(Protocol, 35 January)

Many posts emphasize the importance of maintaining a healthy lifestyle, reflecting on what truly matters in life, and focusing on recovery. Key aspects include eating nutritious food, establishing good sleep routines, and finding a balance between rest and activity. It's also important to remember the consequences of not being able to slow down, which can serve as a friendly reminder to take things at a manageable pace:

Having a stroke increases stress in the body, which can impair brain function, including concentration and memory. The writer also shared links to two articles and a TV show about stress. (Protocol, 63 February)

In those circumstances, it can even be suggested that it is important to monitor oneself to detect if there have been any changes over the last months that might have increased the amount of stress (less sleep, more stress, or drinking alcohol) or if there is a high risk of pushing oneself too much. Even too much exercise becomes unhealthy when it results in overriding one's judgment, which calls for necessary adjustments.

The mentor encourages other members to adapt to the new situation instead of worrying or ruminating. Some mentors even advise people to remove from issues and people that make them worried, such as acquaintances and relatives or demands for a return to working life.

A mentor can provide valuable advice on medication and other care-related issues. Additionally, people often share their own experiences as cautionary examples:

They experienced their first stroke in their late 40s but hesitated to go to the hospital. Despite leading a healthy lifestyle free from alcohol. They urge everyone to seek immediate medical attention if they suspect a stroke since timely intervention is crucial. (Protocol 98, January)

Acting as a mentor also involves sharing own and other experiences of inadequate or unequal care, raising awareness of regional differences regarding the provision of rehabilitation after stroke. In addition, some physicians lack competence regarding the side effects of medicines and knowledge about how different medicines can be combined. In the comments, several people provide examples of medications that have resulted in negative side effects. The critique also underlines the lack of routines, for example, hand-over routines, which jeopardize the health and well-being of patients. In these situations, stroke patients may be at risk of being lost, which puts great responsibility on the person in need of care or their relatives,

The physician who was supposed to provide follow-up care had changed the workplace. Luckily, it is possible to do a self-referral to another clinic. S(he) got a quick response and got a visiting time. (January, protocol, 78)

Exchanging and sharing experiences within the group increases the collective knowledge in the group. The ongoing exchange of experiences has a pedagogical function, which creates opportunities for members to gain knowledge about the responsibilities of different caregivers. It also gives people who suffered from a stroke the opportunity to switch between the patient and mentor role. Being active in the group, that is, answering other members’ questions in a thoughtful, well-informed, and respectful manner renders individual members credibility and gives them the potential to become “a knower” (epistemic subject), and eventually become a mentor.

### Struggler

The struggler has difficulties in coping. It involves experiencing profound despair and hopelessness, rather than demonstrating resilience. For people with lived experiences of stroke struggling can involve coming to terms with who they are and stop from feeling ashamed of the person they have become:

They didn't want to tell people outside their family about their stroke because it's hard to find people who will show consideration when they just want to be left alone. (Protocol 69, January)

Being discontent with oneself indicates that people are struggling to get control over their life situation but experience that they fail to find solutions. It is about being in an uphill battle, feeling like a failure, and concerning that, foremost focusing on things that have been lost, such as the ability to control the body, living with brain fatigue, being dependent on others, experiencing emotional distress, and feeling isolated:

They suffered a stroke a year ago, had visual field loss, dizziness, brain fatigue and poor short-term memory. They are afraid of becoming depressed and wonder why they feel worse than they did right after they had their stroke. (Protocol, 52 January)

As a struggler it is hard to accept that you are not the same person you were before you had a stroke. Some people even experience that the recovery process goes backward:

A person with lived experience of stroke expresses that they had a stroke some years ago and thought that they recovered quickly, but now finds themselves going backward, their motor function has declined and they also find it difficult to read. (Protocol 92, December)

Others experience that although they rest a lot, it is difficult to cope with everyday tasks, and they are devastated about not being able to do chores themselves. It becomes an emotional hardship both for people with lived experience and their close family members and it can result in a lack of self-esteem and feelings of shame and inadequacy.

A relative wrote about a situation where her/his relative had experienced a lot of setbacks:

S(he) worries about her relative, who is going through a separation and changing jobs. S(he) wishes s(he) could make her/his relative feel better. (Protocol 102, January)

A person with lived experience of stroke wonders if other people also can feel depressed:

S(he) does not feel that her/his well-being is being improved by recurring contact with physicians or contact with SSIA because the focus of these meetings is talking about the negative consequences of having a stroke. S(he) often ends up “feeling blue.” (Protocol, 1, May)

Lack of ontological safety is mirrored through expressions such as, “Having the rug pulled under one's feet” indicating that some people who had a stroke live in a constant fear of having yet another one, which may worsen their situation.

### Outsider

Experiencing failure often leads to feelings of being misunderstood and devalued, resulting in a sense of identifying as an outsider. These emotions can create a sense of isolation and frustration, making it difficult to cope and move forward. Feeling like an outsider is related to experiences of not fitting in familiar places and relationships. Misfitting relates to the environment (context, space, and place) as well as interactions with others, where people with lived experience of stroke experience stigma, that is, being misunderstood by others, not being listened to, and experiencing devaluation. Misfitting occurs in the discrepancy between body and world—between that which is expected from others due to social norms but also in relation to one's own ability which makes misfitting both an interpersonal experience and an intra-psychological experience. The outsider is being excluded from social interactions which also contributes to a reduced self-confidence. Not being invited to dinner or other social activities that they used to do together makes people feel excluded and unwelcome.

Experiences of misfitting also occur in relation to being misunderstood. In these situations, people with lived experience of stroke, end up feeling frustrated, experiencing that friends and former colleagues at work think that they are self-absorbed while they struggle to convey their experiences and make themselves understood,

It is hard for someone who has not experienced constant pain to understand what it feels like. It is difficult to understand “hidden impairments” such as pain. It is harder to gain an understanding when one's difficulties are not visible. It is difficult to get people without lived experience to understand what it is like to have suffered a stroke. (Protocol, 7 February)

The outsider expresses feelings of loneliness and isolation, related to being misunderstood and invalidated by others, which also contributes to withdrawing from social gatherings.

Brain fatigue makes them more selective about who they meet. Self-confidence fluctuates, and with each additional diagnosis, a friend may disappear. However, “real friends” remain. Many friendships are lost at the onset of a stroke. (Protocol 1, March)

Experiences of invalidation occur when healthcare professionals question the lived experience of stroke, and in addition, fail to respond to the onset of stroke,

S(he) wishes that healthcare professionals had better knowledge of stroke. S(he) shared a story of being sent home with bleeding in the cerebellum. In the comments, another person wrote about being dismissed without receiving an x-ray, only to return a few days later with more bleeding. (Protocol 150, January)

These types of posts often redefine people's roles as survivors who combat ignorance and lack of knowledge among healthcare staff. This can position them either as strugglers or outsiders, depending on whether they perceive their challenges as specific to certain situations or feel disconnected in all situations. These narratives often go hand in hand with narratives that underline when healthcare staff have been wrong in predictions of physical or cognitive recovery after stroke.

## Discussion

This study explores how individuals with lived experience of stroke construct and negotiate identities in an online peer-support community. By applying Laclau and Mouffe's discourse theory^37^ to FB posts and comments, five recurring subject positions emerged; *Survivor*, *Pathfinder*, *Mentor*, *Struggler*, and *Outsider*. These subject positions reflect how stroke survivors engage in a dynamic, relational process of identity reconstruction, negotiating internal shifts as well as external social expectations. The findings make an important contribution to both discourse analysis and the expanding field of online health support communities.

The identified subject positions reflect a continuum of recovery narratives and discursive responses to the experience of stroke. The *Survivor* and *Mentor* positions correspond closely to “restitution” narratives,^49^ emphasizing the ability to overcome adversity and regain function, purpose, or in some cases employment. These positions align with broader societal discourses that value productivity, independence, and resilience.^51,57^ In contrast, the *Pathfinder* and *Struggler* positions reflect more complex, transitional, or fragmented experiences of recovery. They resonate with “quest” and “chaos” narratives, in which individuals acknowledge enduring losses while attempting to make sense of a changed identity.^45,49^

The *Outsider* position captures the lived consequences of misfitting, not meeting social or professional expectations post-stroke, which highlights the interpersonal and institutional forms of invalidation experienced by survivors. This resembles Garland-Thomson's concept of the “misfit,”^58^ where social environments are not designed to accommodate bodies with impairments, reinforcing marginalization. Posts describing misunderstandings by employers, friends, and healthcare professionals show how these discourses of “misfit” translate into diminished self-worth and increased isolation, also within formal rehabilitation systems.

Taken together, these subject positions demonstrate both the plurality and the fluidity of post-stroke identity processes. The members often moved between these positions depending on changes in health, emotional state, or the nature of interaction within the community. This aligns with discourse theory's emphasis on the contingent nature of subjectivity and the ways in which meaning is negotiated through language and social interaction.^37^ The findings challenge overly linear or universal models of stroke recovery and highlight the value of creating discursive space for diverse, sometimes contradictory, experiences to coexist.

The findings complement and extend prior research on online peer support communities. Consistent with earlier findings,^25,27^ the results demonstrate how the FB group served multiple functions: emotional support, informational exchange, validation, and narrative reconstruction. It was a space for members to seek guidance (*Pathfinders*), express vulnerability (*Strugglers*), or offer mentorship and expertise (*Mentors*). These functions may be particularly crucial for stroke survivors who may lack access to formal psychosocial rehabilitation or who feel alienated from traditional medical discourse.

The findings support the notion that online environments lower psychological and social barriers to disclosure, enabling the expression of sensitive or stigmatized experiences.^19,20^ Members often discussed mental health challenges, dependency, and feelings of shame, topics that by previous studies are described as underacknowledged in face-to-face rehabilitation settings.^19^ Notably, *Strugglers* and *Outsiders* expressed a need to share their sense of loss, fear of recurrence, and struggles with invisible symptoms such as brain fatigue or cognitive impairments, confirming that digital platforms offer unique affordances for marginalized narratives.

Moreover, the findings illustrate how the community functions as a *counter public*, a discursive space in which dominant norms can be challenged or reinterpreted. Members shared critical reflections on healthcare inequalities, misdiagnoses, and the limitations of current rehabilitation practices, suggesting that online communities can foster collective critique and advocacy as well as emotional resilience. This reinforces previous research that such communities can support self-management and influence patients’ interactions with formal care systems.^31^

### Implications for stroke recovery practice

These findings have several implications for practice. The diversity of subject positions illustrates how stroke recovery is not a singular or standardized process, rather shaped by personal biography, social structures, and discursive resources. This has implications on rehabilitation professionals to reconsider purely biomedical or work-oriented measures of recovery and recognize identity work as one of the core dimensions of post-stroke adaptation.

The community's discursive practices illustrate how peer support enables identity reformation through recognition and validation.^36^ Especially for *Pathfinders* and *Strugglers*, being seen and affirmed by others with similar experiences facilitated coping and normalized emotional responses. Integrating structured peer support into formal stroke recovery programs could enhance psychosocial outcomes and promote well-being.

This study highlights a need to re-examine current discourses within policy and rehabilitation frameworks, particularly the emphasis on rapid reintegration into paid employment which risks marginalizing those whose recovery process does not include a return to work.^3^ The findings illustrate the need for more flexible, patient-centered services that allow diverse narratives, including those shaped by long-term disability, emotional trauma, or shifting life priorities.

### Limitations and future research

This study has several limitations. While the data collection period captured a significant volume of posts, the analysis reflects a specific time frame within a developing community. Longitudinal analysis could probably better have captured how discourses and subject positions shift over time and in response to external events such as policy changes or media discourses on stroke. To deepen the understanding of identity construction and discursive practices in digital stroke communities, future research could benefit from a netnographic approach.^59^ Given the current study's focus on a demographic group of primarily Swedish, middle-aged women, future netnographic research could be suitable. Comparing multiple online communities could provide a more nuanced, context-rich, and temporally sensitive understanding of how cultural scripts and societal discourses influence the performance of stroke-related identities.

Finally, this study focused primarily on posts from survivors themselves, with less emphasis on the narratives of family members or caregivers. Given that family often plays a critical role in stroke recovery and identity support, future studies should explore how family members’ discourses interact with or complicate survivor narratives. Despite these limitations, this study extends the understanding of identity construction and stroke recovery.

## Conclusion

The conducted discourse analysis demonstrates that online peer-support communities’ function not only as sources of emotional and practical help, but as sites of identity negotiation and discursive empowerment. The identified subject positions reveal how individuals with lived experience of stroke construct meaning, resist stigmatizing discourses, and support each other through evolving narratives of recovery. By engaging with these identities, members develop a shared language of resilience, critique, and belonging. This language both complements and challenges traditional medical discourses. The findings suggest a broadening of the conception of stroke recovery among health care practitioners and policymakers, to include narrative flexibility, emotional care, and social connectedness. Peer-led online communities seem to offer a promising model for fostering these dimensions and may be recognized as a valuable part of the long-term recovery landscape.

## Research team

Lill Hultman (LH), PhD, Associate Professor Social Work, researcher; Ulla-Karin Schön, (UKS), PhD, Professor Social Work, researcher; Fredrik Sandman (FS), Community researcher, Mikael Åkerlund (MÅ), Community researcher; Jeanette Nelson, (JN), Community researcher; Malin Tistad, (MT), MD, Associate Professor Care Sciences, researcher.

## Supplemental Material

sj-docx-1-dhj-10.1177_20552076251376274 - Supplemental material for Facebook as an arena for peer support?—Knowledge exchange and normative illness narratives about stroke Supplemental material, sj-docx-1-dhj-10.1177_20552076251376274 for Facebook as an arena for peer support?—Knowledge exchange and normative illness narratives about stroke by Lill Hultman, Ulla-Karin Schön, Fredrik Sandman, Mikael Åkerlund, Jeanette Nelson and Malin Tistad in DIGITAL HEALTH

sj-pdf-2-dhj-10.1177_20552076251376274 - Supplemental material for Facebook as an arena for peer support?—Knowledge exchange and normative illness narratives about stroke Supplemental material, sj-pdf-2-dhj-10.1177_20552076251376274 for Facebook as an arena for peer support?—Knowledge exchange and normative illness narratives about stroke by Lill Hultman, Ulla-Karin Schön, Fredrik Sandman, Mikael Åkerlund, Jeanette Nelson and Malin Tistad in DIGITAL HEALTH

## References

[1] Ljuslinder K Ellis K Vikström L . Cripping time – understanding the life course through the Lens of ableism. Scand J Disabil Res 2020; 22: 35–38.

[2] Elder GH Jr Johnson MK Crosnoe R . The emergence and development of life course theory. In: Mortimer J Shanahan M (eds) Handbook of the lifecourse. New York: Plenum, 2023, pp.3–22.

[3] Maaijwee N Rutten-Jacobs L Schaapsmeerders P , et al. Ischaemic stroke in young adults: risk factors and long-term consequences. Nat Rev, Neurol 2014; 10: 315–325. 24776923 10.1038/nrneurol.2014.72

[4] Wolf T Barbee A White D . Executive dysfunction immediately after mild stroke. OTJR: Occup, Particip Health 2011; 31: S23–S29. 10.3928/15394492-20101108-05 24650260

[5] Röding J Lindström B Malm J , et al. A “frustrated” and invisible-younger stroke patients’ experiences of the rehabilitation process. Disabil Rehabil 2003; 25: 867–874. 12851097 10.1080/0963828031000122276

[6] Cowdell F Garrett D . Recreation in stroke rehabilitation part two: exploring patients’ views. Int J Ther Rehabil 2003; 10: 456–462.

[7] Bendz M . The first year of rehabilitation after a stroke—from two perspectives. Scand J Caring Sci 2003; 17: 215–222. 12919455 10.1046/j.1471-6712.2003.00217.x

[8] Greenwood N Holley J Ellmers T , et al. Qualitative focus group study investigating experiences of accessing and engaging with social care services: perspectives of carers from diverse ethnic groups caring for stroke survivors. BMJ Open 2016; 6: e009498. 10.1136/bmjopen-2015-009498 PMC4735197 26826148

[9] Martinsen R Kirkevold M Bronken B , et al. Work-aged stroke survivors’ psychosocial challenges narrated during and after participating in a dialogue-based psychosocial intervention: a feasibility study. BMC Nurs 2013; 12: 1–10. 24066840 10.1186/1472-6955-12-22 PMC3849869

[10] Essue B Hackett M Li Q , et al. How are household economic circumstances affected after a stroke? The psychosocial outcomes in StrokE (POISE) study. Stroke 2012; 43: 3110–3113.. 22949473 10.1161/STROKEAHA.112.666453

[11] Guise J McKinlay A Widdicombe S . The impact of early stroke on identity: a discourse analytic study. Health 2010; 14: 75–90. 20051431 10.1177/1363459309347483

[12] Bendz M . Rules of relevance after a stroke. Soc Sci Med 2000; 51: 713–723. 10975231 10.1016/s0277-9536(99)00486-4

[13] Strand M Eng LS Gammon D . Combining online and offline peer support groups in community mental health care settings: a qualitative study of service users’ experiences. Int J Ment Health Syst 2020; 14: 39.. PMID: 32514303; PMCID: PMC7260836. 32514303 10.1186/s13033-020-00370-x PMC7260836

[14] Clark E MacCrosain A Ward NS , et al. The key features and role of peer support within group self-management interventions for stroke? A systematic review. Disabil Rehabil 2020; 42: 307–316. 30325686 10.1080/09638288.2018.1498544

[15] Wijekoon S Wilson W Gowan N , et al. Experiences of occupational performance in survivors of stroke attending peer support groups. Can J Occup Ther 2020; 87: 173–181. 32115988 10.1177/0008417420905707

[16] Christensen ER Golden SL Gesell SB . Perceived benefits of peer support groups for stroke survivors and caregivers in Rural North Carolina. N C Med J 2019; 80: 143–148. 31072940 10.18043/ncm.80.3.143 PMC6730634

[17] Yeo G Fortuna KL Lansford JE , et al. The effects of digital peer support interventions on physical and mental health: a review and meta-analysis. Epidemiol Psychiatr Sci 2025; 34: e9. 10.1017/S2045796024000854 PMC11886969 39945388

[18] Duggan M Lenhart A Lampe C , et al. Parents and Social Media. Mothers are especially likely to give and receive support on social media. Pew Research Center. https://www.pewresearch.org/internet/2015/07/16/parents-and-social-media (accessed 25 June 2018)

[19] Wright KB . Communication in health-related online support groups/communities: a review of research on predictors of participation applications of social support theory, and health outcomes. Rev Commun Res 2016; 4: 65–87.

[20] Berger M Wagner TH Baker LC . Internet use and stigmatized illness. Soc Sci Med 2005; 61: 1821–1827.. Epub 2005 Apr 26. PMID: 16029778. 16029778 10.1016/j.socscimed.2005.03.025

[21] Cline RJ Haynes KM . Consumer health information seeking on the internet: the state of the art. Health Educ Res 2001; 16: 671–692.. PMID: 11780707. 11780707 10.1093/her/16.6.671

[22] Highton-Williamson E Priebe S Giacco D . Online social networking in people with psychosis: a systematic review. Int J Soc Psychiatry 2015; 61: 92–101.. Epub 2014 Nov 6. PMID: 25381145. 25381145 10.1177/0020764014556392

[23] Smith-Merry J Goggin G Campbell A , et al. Social connection and online engagement: insights from interviews with users of a mental health online forum. JMIR Ment Health 2019; 6: e11084.. PMID: 30912760; PMCID: PMC6454344. 10.2196/11084 PMC6454344 30912760

[24] Naslund JA Aschbrenner KA Marsch LA , et al. The future of mental health care: peer-to-peer support and social media. Epidemiol Psychiatr Sci 2016; 25: 113–122.. Epub 2016 Jan 8. PMID: 26744309; PMCID: PMC4830464. 26744309 10.1017/S2045796015001067 PMC4830464

[25] Barak A Boniel-Nissim M Suler J . Fostering empowerment in online support groups. Comput Human Behav 2008; 24: 1867–1883.

[26] Nilsson S Hård af Segerstad Y Olsson M . Visualizing the invisible-the needs and wishes of childhood cancer survivors for digitally mediated emotional peer support. Curr Oncol 2022; 29: 1269–1278. 35200607 10.3390/curroncol29020108 PMC8870810

[27] Lawless MT Hunter SC Pinero de Plaza MA , et al. You are by no means alone: a Netnographic study of self-care support in an online community for older adults. Qual Health Res 2022; 32: 1935–1951. 36062369 10.1177/10497323221124979

[28] Prescott J Rathbone AL Brown G . Online peer to per support: Qualitative analysis of UK and US open mental Facebook groups. Digit Health 2020: 6: 1–17. 10.1177/2055207620979209 PMC7734541 33354335

[29] Liu Y Kornfield R Shaw BR , et al. When support is needed: Social support solicitation and provision in an online alcohol use disorder forum. Digit Health 2017; 3: 1–16. 10.1177/2055207617704274 PMC6001259 29942595

[30] Thorn P La Sala L Hetrick S , et al. Motivations and perceived harms and benefits of online communication about self-harm: an interview study with young people. Digit Health 2023; 9: 1–13. 10.1177/20552076231176689 PMC10214072 37252260

[31] Joglekar S Sastry N Coulson NS , et al. How online communities of people with long-term conditions function and evolve: network analysis of the structure and dynamics of the asthma UK and British lung foundation online communities. J Med Internet Res 2018; 20: e238. 10.2196/jmir.9952 PMC6060304 29997105

[32] White M Epston D . Narrative means to therapeutic ends. New York: Norton, 1990.

[33] Antaki C . Explanations, communication and social cognition. In: Antaki C (ed.) Analysing everyday explanation: a casebook of methods. London: Sage Publications, Inc, 1988, pp.1–14.

[34] Stainton Rogers W. (1991). Explaining health and illness ‒ an exploration of diversity. New York, London: Harvester Wheatsheaf.

[35] Cohler BJ . Personal narrative and life course. In: Baltes P Brim OG (eds) Life span development and behavior. 4. New York, NY: Academic Press, 1982, pp.205–241.

[36] Gergen MM . Self-narration in social life. In: Wetherell M Yates STSJ (eds) Discourse theory and practice. London: The open University: Sage, 2001, pp.247–260.

[37] Laclau E Mouffe C. Hegemony and socialist strategy: Towards a radical democratic politics. London: Verso, 2001.

[38] Winther Jørgensen M Phillips L. Diskursanalys som teori och metod. Lund: Studentlitteratur, 2000.

[39] Börjesson M Palmblad E . Diskursanalys i praktiken. Malmö: Liber, 1995.

[40] Bergström G Boréus K . Samhällsvetenskaplig text-och diskursanalys. In: Boréus K Bergström G (eds) Textens mening och makt: metodbok i samhällsvetenskaplig text-och diskursanalys. 4th ed. Lund: Studentlitteratur, 2018, pp.17–48.

[41] Bury MR . Chronic illness as biographical disruption. Sociol Health Illn 1982; 4: 167–182. 10260456 10.1111/1467-9566.ep11339939

[42] Kaufman S Becker G . Stroke: health care on the periphery. Soc Sci Med 1986; 22: 983–989.. PMID: 3526567. 3526567 10.1016/0277-9536(86)90171-1

[43] Faircloth CA Boylstein C Rittman M , et al. Constructing the stroke: sudden onset narratives of stroke survivors. Qual Health Res 2005; 2005: 928–941. 10.1177/1049732305277842 16093371

[44] Tosi MT . Personal identity and moral discourse in psychotherapy. Transact Anal J 2018; 48: 139–151.

[45] Tosi MT . The lived and narrated script: an ongoing narrative construction. In: Erskine RG (eds) Life scripts: a transactional analysis of unconscious relational patterns. London: Karnac Books, 2010, pp.29–54.

[46] Corbin JM Strauss A . A nursing model for chronic illness management based upon the trajectory framework. In: Woog P (eds) The chronic illness trajectory framework. New York: Springer, 1992, pp.9–28.

[47] Burton CR . Re-thinking stroke rehabilitation: the Corbin and Strauss chronic illness trajectory framework. J Adv Nurs 2000; 32: 595–602. 11012801 10.1046/j.1365-2648.2000.01517.x

[48] Frank AW. The wounded storyteller. Chicago and London: The University of Chicago Press, 1995.

[49] Campbell J . The hero with a thousand faces. Princeton, NJ: Princeton University, 2008.

[50] Giddens A. Modernity and self-identity: self and society in the late modern age. Cambridge: Polity Press, 1991, pp.10–34.

[51] Van Houten D Jacobs G . The empowerment of marginals: strategic paradoxes. Disabil Soc 2025; 6: 641–654.

[52] SOU 1988:41 . *Tidig och samordnad rehabilitering. Samverkansmetoder och rehabiliteringsinriktad ersättning, m.m* *.*

[53] Bergman J Wall E . Ålder och funktionalitet: om vuxna barns ansvarstagande för ensamboende föräldrars trygghet. In: Krekula C Johansson B (eds) Introduktion till kritiska åldersstudier. Lund: Studentlitteratur, 2017, pp.183–196.

[54] Lövgren V . De sociala konstruktionerna av hög ålder och funktionsförmåga–exemplet intellektuellt funktionshinder. Sociol Forsk 2014; 51: 47–64.

[55] Harvey D . A brief history of neoliberalism. New York: Oxford University Press, 2005.

[56] Kaae Kristensen H Præstegaard J Ytterberg C . Discourses in stroke rehabilitation as they present themselves in current physiotherapy and occupational therapy. Disabil Rehabil 2017; 39: 223–235. 26878167 10.3109/09638288.2016.1138554

[57] Johansson A. Självskada. En etnologisk studie av mening och identitet i berättelser om skärande . PhD Thesis. Etnologiska skrifter 51. Institutionen för Kultur och Mediaskapande, Umeå University, Sweden. 2010.

[58] Garland-Thomson RM. Misfit- A feminist materialist disability concept. Hypatia 2011; 23: 453–666.

[59] Kozinets RV . Netnography. Doing ethnographic research online. Los Angeles, CA: University of Southern California, 2010.

