# Supplementary material for: Facebook as an arena for peer support?—Knowledge exchange and normative illness narratives about stroke
Source: Digit Health. 2025 Sep 9;11:20552076251376274. doi: 10.1177/20552076251376274 (PMC12420960; doi:10.1177/20552076251376274)
Supplement: sj-docx-1-dhj-10.1177_20552076251376274 - Supplemental material for Facebook as an arena for peer support?—Knowledge exchange and normative illness narratives about stroke [file sj-docx-1-dhj-10.1177_20552076251376274.docx]

**Name of peer-to-peer online support group**

**Date of data collection:**

**Date the post was written:**

**Data collection made by:**  Jeanette  Fredrik  Micke  Lill Malin

**Who wrote the post?**

Person with lived experience of stroke

Close relative (husband, wife, significant other, child, parent)

Administrator

Not specified

Other (specify): __ _________________________

**Gende of the person who wrote the post?**

Man

Woman

Not specified

**Who is the post about?**

Person with lived experience of stroke as an individual

Person with lived experience of stroke as a group

Close relatives (husband, wife, significant other, child, parent) as individuals

Close relatives (husband, wife, significant other, child, parent) as a group

Not specified

Other (specify): __________

**Type of post:**

Question

Story

Call for action

Inspirational quote/poetry

Picture or photograph

Sharing a link

Invitation to an event

Other (specify):

| **Information about the post** | |
| --- | --- |
| **Brief description of the content of the post** |  |
| **Description of any pictures or photographs** |  |

| **Comments to the post** | |
| --- | --- |
| **Summary of comments** |  |
| **Amount of comments**  **made by men and women** |  |
| **Amount of ‘likes’ the post has received** |  |
| **Number of times a post has been shared** |  |
| **Reflections about the post made by the data collector** |  |
